# Case of Dry Mortification, Preceded by Convulsions, in a Puerperal Patient

**Published:** 1845-01

**Authors:** G. N. Fitch

**Affiliations:** Professor of Obstetrics, &c., in the Rush Medical College; Logansport, Ind.


### *Case of Dry Mortification, preceded by Convulsions, in a Puerperal Patient.* By G. N. Fitch, M.D., Professor of Obstetrics, &c., in the Rush Medical College.

Mrs. K****, aged 18, of full habit, florid countenance, and enjoying good health, in labor with her first child, (in the summer of 1843,) was seized with violent convulsions. Being present, I bled her copiously. Relief from the convulsive movements and evidences of cerebral determination immediately followed, but not a return of sensibility. A blister was applied to the back of the neck, an enema administered, followed by an active saline cathartic. Uterine efforts continued, but the fœtus not being sufficiently low in the pelvis to safely permit prompt manual, or instrumental delivery, the case was left to its natural progress. Delivery, of a dead child, occurred in about six hours after the bleeding. Complete sensibility was restored in about the same length of time after delivery, and the patient bade fair for a rapid and entire recovery of health and strength.

The third morning after delivery, however, a messenger informed me that she had suffered all the previous night with violent pain in the great toe of the left foot. Surrounded by a press of professional engagements, I paid but little attention to the report, presuming the pain a trivial matter, consequent, perhaps, upon some injury received during the convulsion. An opiate liniment was prescribed. The next day the pain was represented as so excruciatingly severe, I visited the patient. She was in bed, in a sitting posture, grasping the affected foot, a short distance above the toe, with both hands; her countenance pale, and indicative of great distress, pulse accelerated, weak and irritable, tongue moist and but slightly coated, no thirst, and appetite good. Surface of natural temperature, except that of the affected foot, in which there appeared to be an entire absence of animal heat. No tumefaction, no discoloration, but a deadly coldness of the foot, and exquisite tenderness of the painful toe. The severity of the pain equalled that of any case which has fallen under my observation.

The pulse and previous depletion, for convulsions, forbade the use of the lancet. Local applications of every grade of stimulation and temperature were unproductive of any mitigation of the severe suffering, except an infusion of hops in vinegar, which usually procured a short interval, not of exemption from pain, but mitigation of its utmost severity. Opiates became the only resource, and were freely administered. On the fifth day from the commencement of pain, a small dark-brown spot presented itself on the upper surface of the great toe. This spread rapidly, and as it extended, the pain, equally severe, became more diffuse, occupying the surface immediately contiguous to the gangrenous. There was no alteration in the color or temperature of the sound part, but an absence of the extreme tenderness to the touch which previously existed in the toe. The gangrenous part had a dark-brown, dry, shrunken, or shrivelled appearance; there was no discharge, no fœtor, and no detachment of the cuticle, except in one place, where a small blister had been applied (without my advice) upon the then sound part, but which the gangrene entirely occupied the next day. I have said there was "no discharge, no fœtor." Such was the case, until the disease occupied about one-third of the foot, when a partial line of separation between the dead and living parts became manifest on the outer side of the foot. From this line issued a thin sero-purulent discharge, extremely offensive. Meantime the disease was still spreading on the internal side of the foot, with no mitigation of pain. The patient, at times delirious, was rapidly sinking from loss of sleep, recent loss of appetite, and offensive discharge. It was evident she could not survive long enough to permit a natural separation of the dead part. Amputation above the ankle, as high up as the *extreme* coldness of surface extended, was proposed. It was preferred (by the husband and the father) that the operation should be performed through the tarsus, even at the risk of her being compelled to submit to it, a second time, in consequence of the disease seizing the stump. The tourniquet having been applied, the amputation was commenced through the articulation of the scaphoid and astragalus, and completed by sawing through the cuboid. In its progress, discovering there was no hæmorrhage, the tourniquet was taken off. Not a spoonful of blood was lost and no vessel tied. The stump was dressed in other respects as usual. The patient improved rapidly, and the stump entirely healed in about two weeks. She has since had a premature birth of twins, (dead born). Her general health is good, but the entire lower half of that limb unnaturally cold.

What was the cause of the gangrene? Most assuredly not ergot She had taken none medicinally, the family consumed no rye, and the patient ate very little bread of any kind, except from corn. Was it connected with the previous convulsions? Or arising from ossification or inflammation of the arteries? The latter, from the symptoms, is the most probable cause. It appears, however, to have formed an exception to the general rule in such cases,—not to amputate, before a well-marked line exists between the dead and living parts.

Logansport, Ind., Nov., 1844.

